# Mass Spectrometric Proteomics 2022

**DOI:** 10.3390/ijms232214246

**Published:** 2022-11-17

**Authors:** Paolo Iadarola

**Affiliations:** Department of Biology and Biotechnologies “L.Spallanzani”, University of Pavia, Via A.Ferrata 9, 27100 Pavia, Italy; piadarol@unipv.it

Until recently, a major challenge of biochemists working in the protein field was the identification, purification, and sequencing of an individual protein. With the advent of proteomics, the identification and quantification of nearly all proteins expressed in a biological system, i.e., the proteome, is achievable in a single experiment. This is possible thanks to the advancements of technologies that have pushed the boundary of research towards increasingly higher goals and allow the obtainment of a global and integrated view of biological questions investigating the entire set of proteins of a cell rather than each one individually. Proteomics may provide information not only on the number and abundance of proteins expressed in a system but also on their involvement in metabolic pathways, their post-translational modifications, interaction, movement between subcellular compartments, and their rates of production and degradation. The dynamics of the proteome over time are of particular relevance. On the assumption that proteome changes reflect, to some degree, modifications occurring in the cell in response to different stimuli, investigation into protein modulation between different conditions may shed light on the biological mechanism involved in these processes. Given the inherently complex nature of cellular proteomes, sophisticated techniques are needed to separate the mixture before analysis. In this regard, the full benefits of electrophoretic/chromatographic applications for the separation of very complex mixtures with high speed, sensitivity, and great resolution are well documented by articles in which high-performance liquid chromatography (HPLC) and ultra-high performance liquid chromatography (UHPLC) show their potential. A crucial role is then played by mass spectrometric (MS) methods with their ability to identify proteins and address an ever-increasing array of biological questions including measuring dynamic changes in protein expression, modification, and interaction. The intention of this Special Issue is to highlight the results obtained by applying sophisticated methodological strategies to profile proteins from different biological fluids and cells in the investigation of the molecular mechanisms behind different human disorders. The first article, by Diaz-Riera et al. [1], clearly shows that, far from being outdated, two-dimensional electrophoresis (2-D) coupled to matrix-assisted laser desorption ionization time-of-flight (MALDI-TOF/TOF) is a technique that is still very helpful for gaining an understanding of the pathophysiology of a disease. The analyses of urine samples from patients with acute decompensated hearth failure (ADHF) vs. samples from healthy subjects revealed the presence of 26 differential proteins that could trace a link with disease evolution. Retinol metabolism and transport, immune response/inflammation, extracellular matrix organization, and platelet degranulation were the top four biological pathways to which the differential proteins belonged. In particular, two of them, that is, transthyretin in combination with retinol-binding protein 4, were identified as specific urine protein signatures in ADHF at hospitalization. Aiello et al. [2] adopted a 3D scaffold-free spheroid model of primary dermal fibroblasts to investigate the mechanism of action of carnosine on the skin proteome of a 50-year-old donor. The label-free proteomics approach they applied allowed the in-depth characterization of the 3D dermis phenotype during growth and differentiation, at 14 vs. 7 days of culture, through the quantitation of 2171 proteins, 485 of which were differentially regulated by carnosine at 7 days. From among the several modulated pathways, most (i.e., oxidative phosphorylation, TCA cycle, extracellular matrix reorganization and apoptosis) were involved in mitochondrial functionality. The work of Grangeon et al. [3] focused on the expression levels of the members of the CYP450 superfamily that are expected to contribute to pre-systemic biotransformation of drugs in the human small intestine. They applied UHPLC-MS targeted proteomics for the quantitation of expression levels of 16 major CYP450 isoforms in subsections (duodenum, jejunum, and ileum) of the small intestine obtained from nine human donors. Detection and quantification in various sections of the small intestine of seven potential CYP450 isoforms provided new data on the absolute quantification of CYP450 in the human small intestine that could be informative about drug absorption profiles and improve pharmacokinetic models. UHPLC coupled to TOF-MS was also used by Schinagl et al. [4] to provide an update on the characteristics of hepatic stellate cells (HSC) activation. They exposed immortalized human LX-2 HSC to either 1% or 10% foetal bovine serum (FBS). Quiescent (1% FBS) and activated (10% FBS) LX-2 cells resulting from this transformation were then subjected to in-depth MS-based analysis and comprehensive phenotyping. An increase in the production of ribosomal proteins and proteins related to cell-cycle control and migration (resulting in higher proliferation and faster migration phenotypes) and a decrease in the expression of proteins related to cholesterol and fatty acid biosynthesis, together with the loss of cytosolic lipid droplets during activation, were observed from protein network analysis of activated LX-2 cells. Burat et al. [5] explored by UHPLC-MS to the potential of shotgun proteomics in the characterization of human eccrine sweat as a bio-fluid of interest for diagnosis and personalized therapy. The analysis of samples from 28 healthy adult volunteers enabled the identification of 983 unique proteins, 344 of which were found in all samples. Interestingly, despite the high similarity between sweat proteomes of females and males, gender-exclusive proteins and gender-specific protein abundances were observed. The fact that the proteins identified were involved in many diverse biological processes and molecular functions (actin dynamics, oxidative stress, proteolysis, anti-microbial immunity, and proteasome-related functions) indicated the potential of this bio-fluid as a valuable biological matrix for further studies. The work of Ponzini et al. [6] focuses on the protein content of the lacrimal fluid. They applied ultrahigh-resolution shotgun proteomics (LC-MS/MS) to perform the analysis of single-tears from 23 healthy human volunteers. This procedure allowed the identification, with high confidence, of a total of 890 proteins whose hierarchical clustering revealed the possibility to stratify females vs. males. This result had never emerged from previous studies on pooled samples. The use of a very sophisticated technology known as multidimensional protein-identification technology (MudPIT) combined with bioinformatic tools allowed De Palma et al. [7] to generate, for the first time, the whole protein profile of lymphoblastoid cells from subjects affected by Nasu–Hakola disease (NHD). This recessively inherited systemic leukodystrophy disorder is characterized by frontotemporal presenile dementia combined with lytic bone lesions. The application of this fully automated platform to lymphoblastoid cells of individuals affected by NHD, healthy carriers and control subjects belonging to the same family allowed the identification of about 3000 distinct proteins within the cohorts investigated. Among these, proteins specific for each category were identified and several differentially expressed proteins could be associated with neurodegenerative processes. Moreover, the analysis of protein networks highlighted some molecular pathways that may be involved in the onset or progression of this rare frontotemporal disorder. The article by D’Amato et al. [8] concludes the review of the reports present in this Special Issue. It consists of a review article focused on the application of proteomic techniques to sputum of patients affected by severe lung disorders such as COPD, asthma, cystic fibrosis, lung cancer, and those caused by COVID-19 infection. By exploring the state-of-the-art over the period 2011–2021, it can be inferred that sputum is an important source of information, the level of several proteins identified in the works considered being changed in response to the organism’s condition. This is very important since understanding the proteome dynamism may allow the association of these proteins with alterations in the physiology or progression of diseases investigated.
